# Bis{μ-1,3-bis­[(2-methyl-1*H*-benzimid­azol-1-yl)meth­yl]benzene-κ^2^ *N* ^3^:*N* ^3′^}bis­(diiodidocadmium)

**DOI:** 10.1107/S1600536811042334

**Published:** 2011-10-22

**Authors:** Jiyong Hu, Junhong Liu, Jin’an Zhao

**Affiliations:** a Department of Chemistry and Chemical Engineering, Henan University of Urban Construction, Henan 467036, People’s Republic of China; b Department of Bioengineering, Henan University of Urban Construction, Henan 467036, People’s Republic of China

## Abstract

In the title compound, [Cd_2_I_4_(C_24_H_22_N_4_)_2_], the 1,3-bis­[(2-methyl-1*H*-benzimidazol-1-yl)meth­yl]benzene ligand bridges two CdI_2_ units, forming a centrosymmetric dinuclear complex. The Cd^II^ atom adopts a distorted tetra­hedral coordination geometry. In the crystal, complex mol­ecules are linked into columns parallel to [101] by π–π stacking inter­actions, with centroid–centroid distances of 3.558 (2) Å.

## Related literature

For general background to the synthesis and properties of benzimidazole metal complexes, see: Wang *et al.* (2006 ▶); Yu *et al.* (2010 ▶); Li *et al.* (2011 ▶); Dobrzanska *et al.* (2006 ▶). For related structures, see: Raehm *et al.* (2003 ▶); Zhao *et al.* (2009 ▶).
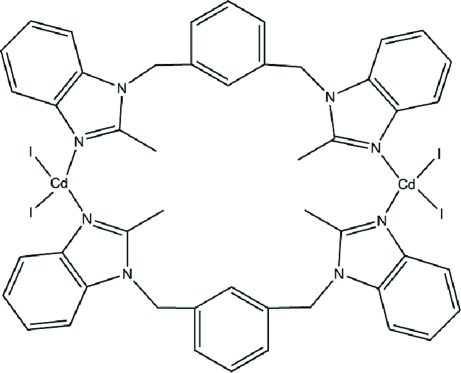


## Experimental

### 

#### Crystal data

[Cd_2_I_4_(C_24_H_22_N_4_)_2_]
*M* *_r_* = 1465.33
Triclinic, 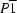

*a* = 9.3968 (19) Å
*b* = 11.286 (2) Å
*c* = 11.703 (2) Å
α = 87.20 (3)°
β = 84.60 (3)°
γ = 86.03 (3)°
*V* = 1231.5 (4) Å^3^
*Z* = 1
Mo *K*α radiation
μ = 3.41 mm^−1^
*T* = 293 K
0.20 × 0.10 × 0.08 mm

#### Data collection

Rigaku Saturn 724 CCD diffractometer
Absorption correction: multi-scan (*CrystalClear*; Rigaku/MSC, 2006 ▶) *T*_min_ = 0.549, *T*_max_ = 0.772
13506 measured reflections
4836 independent reflections
4153 reflections with *I* > 2σ(*I*)
*R*_int_ = 0.028

#### Refinement

*R*[*F* ^2^ > 2σ(*F* ^2^)] = 0.037
*wR*(*F* ^2^) = 0.080
*S* = 1.05
4836 reflections
282 parameters
H-atom parameters constrained
Δρ_max_ = 0.79 e Å^−3^
Δρ_min_ = −1.22 e Å^−3^

### 

Data collection: *CrystalClear* (Rigaku/MSC, 2006 ▶); cell refinement: *CrystalClear*; data reduction: *CrystalClear*; program(s) used to solve structure: *SHELXS97* (Sheldrick, 2008 ▶); program(s) used to refine structure: *SHELXL97* (Sheldrick, 2008 ▶); molecular graphics: *CrystalStructure* (Rigaku/MSC, 2006 ▶); software used to prepare material for publication: *CrystalStructure*.

## Supplementary Material

Crystal structure: contains datablock(s) I, global. DOI: 10.1107/S1600536811042334/rz2634sup1.cif

Structure factors: contains datablock(s) I. DOI: 10.1107/S1600536811042334/rz2634Isup2.hkl

Additional supplementary materials:  crystallographic information; 3D view; checkCIF report

## Figures and Tables

**Fig. 1.**
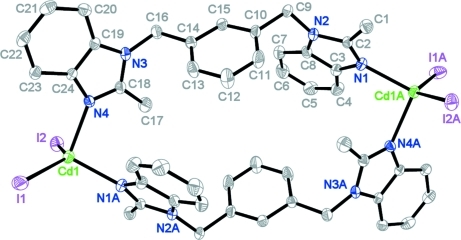
The molecular structure of the title complex, showing 30% probability displacement ellipsoids. Hydrogen atoms are omitted for clarity. Atoms labelled with suffix A are generated by the symmetry operation (1-x, -y, 1-z).

## supplementary crystallographic information

### Comment

Metallamacrocycle species possess cavities whose size can be readily modified for selective encapsulating properties and other functionalities. Among others, benzimidazole and its derivatives have become promising building blocks resulting from their wide-ranging biological activities, interesting photochemical and photophysical properties, versatile coordination modes according to the different geometric requirements of metal centers, and potential ability to form supramolecular aggregates with unique structural topologies and interesting properties through π–π aromatic stacking and hydrogen-bonding interactions (Wang *et al.*, 2006; Yu *et al.*, 2010; Li *et al.*, 2011; Dobrzanska *et al.*, 2006).

The asymmetric unit of the title compound consists of a CdI_2_ unit and a 2-methyl-1*H*benzimidazol-1-yl)methyl]benzene molecule, where the ligand bridges two metal atoms forming a centrosymmetric dinuclear complex molecule (Fig. 1). The separation between the metal atoms is 13.373 (4) Å, and the potential accessible volume estimated by *PLATON* (Spek, 2009) is 3.9% of the total crystal volume. The dihedral angles formed by the benzene ring with the benzimidazole rings are 74.73 (13) and 82.56 (14)°. Because of the presence of the methyl groups and coordination requirement of the metal, the ligand assumes a remarkably different conformation with respect to those observed in the related Zn (Zhao *et al.*, 2009) and Ag (Raehm *et al.*, 2003) dinuclear complexes. In the crystal packing, complex molecules are interact through π–π stacking interactions to form into columns parallel to the [101] direction (Cg1···Cg1^i^ = 3.558 (2) Å; Cg1 is the centroid of the C19–C24 ring; symmetry code: (i) -x, -y, -z).

### Experimental

To a solution of CdI_2_ (0.02 mmol, 0.0073 g) in methanol (5 ml) an equivalent amount of the ligand 2-methyl-1*H*-benzimidazol-1-yl)methyl]benzene in DMF (1 ml) was added. After three weeks, stick-shaped colourless crystals were obtained on slow evaporation of the solvents at room temperature.

### Refinement

H atoms were positioned geometrically and refined using a riding model, With C–H = 0.93 Å (CH), 0.97 Å (CH_2_), 0.96 Å (CH_3_), and with *U*_iso_ = 1.2 *U*_eq_(C) or 1.5 *U*_eq_(C) for methyl H atoms.

### Crystal data

**Table:**

[Cd_2_I_4_(C_24_H_22_N_4_)_2_]	*Z* = 1
*M**_r_* = 1465.33	*F*(000) = 696
Triclinic, *P*1	*D*_x_ = 1.976 Mg m^−^^3^
Hall symbol: -P 1	Mo *K*α radiation, λ = 0.71073 Å
*a* = 9.3968 (19) Å	Cell parameters from 7057 reflections
*b* = 11.286 (2) Å	θ = 2.2–26°
*c* = 11.703 (2) Å	µ = 3.41 mm^−^^1^
α = 87.20 (3)°	*T* = 293 K
β = 84.60 (3)°	Stick, colourless
γ = 86.03 (3)°	0.20 × 0.10 × 0.08 mm
*V* = 1231.5 (4) Å^3^	

### Data collection

**Table:**

Rigaku Saturn 724 CCD diffractometer	4836 independent reflections
Radiation source: fine-focus sealed tube	4153 reflections with *I* > 2σ(*I*)
graphite	*R*_int_ = 0.028
dtprofit.ref scans	θ_max_ = 26.0°, θ_min_ = 2.2°
Absorption correction: multi-scan (*CrystalClear*; Rigaku/MSC, 2006)	*h* = −11→11
*T*_min_ = 0.549, *T*_max_ = 0.772	*k* = −13→13
13506 measured reflections	*l* = −14→13

### Refinement

**Table:**

Refinement on *F*^2^	Primary atom site location: structure-invariant direct methods
Least-squares matrix: full	Secondary atom site location: difference Fourier map
*R*[*F*^2^ > 2σ(*F*^2^)] = 0.037	Hydrogen site location: inferred from neighbouring sites
*wR*(*F*^2^) = 0.080	H-atom parameters constrained
*S* = 1.05	*w* = 1/[σ^2^(*F*_o_^2^) + (0.0315*P*)^2^ + 1.6758*P*] where *P* = (*F*_o_^2^ + 2*F*_c_^2^)/3
4836 reflections	(Δ/σ)_max_ < 0.001
282 parameters	Δρ_max_ = 0.79 e Å^−^^3^
0 restraints	Δρ_min_ = −1.22 e Å^−^^3^

### Special details

**Table:**

Geometry. All e.s.d.'s (except the e.s.d. in the dihedral angle between two l.s. planes) are estimated using the full covariance matrix. The cell e.s.d.'s are taken into account individually in the estimation of e.s.d.'s in distances, angles and torsion angles; correlations between e.s.d.'s in cell parameters are only used when they are defined by crystal symmetry. An approximate (isotropic) treatment of cell e.s.d.'s is used for estimating e.s.d.'s involving l.s. planes.
Refinement. Refinement of *F*^2^ against ALL reflections. The weighted *R*-factor *wR* and goodness of fit *S* are based on *F*^2^, conventional *R*-factors *R* are based on *F*, with *F* set to zero for negative *F*^2^. The threshold expression of *F*^2^ > σ(*F*^2^) is used only for calculating *R*-factors(gt) *etc*. and is not relevant to the choice of reflections for refinement. *R*-factors based on *F*^2^ are statistically about twice as large as those based on *F*, and *R*- factors based on ALL data will be even larger.

### Fractional atomic coordinates and isotropic or equivalent isotropic displacement parameters (Å^2^)

**Table:**

	*x*	*y*	*z*	*U*_iso_*/*U*_eq_	
I1	0.09798 (4)	−0.51952 (3)	0.15611 (4)	0.06468 (14)	
I2	0.46486 (4)	−0.30519 (3)	−0.08378 (3)	0.05247 (11)	
Cd1	0.29674 (3)	−0.35597 (3)	0.11355 (3)	0.03755 (10)	
N1	0.5844 (4)	0.3627 (3)	0.7254 (3)	0.0343 (8)	
N2	0.4245 (4)	0.3662 (3)	0.5973 (3)	0.0355 (8)	
N3	0.1485 (4)	0.0178 (3)	0.1876 (3)	0.0338 (8)	
N4	0.1853 (4)	−0.1711 (3)	0.1419 (3)	0.0325 (8)	
C1	0.3315 (5)	0.4102 (4)	0.7993 (4)	0.0426 (11)	
H1A	0.3686	0.4039	0.8732	0.064*	
H1B	0.2567	0.3566	0.7982	0.064*	
H1C	0.2936	0.4902	0.7848	0.064*	
C2	0.4477 (4)	0.3794 (4)	0.7095 (4)	0.0313 (9)	
C3	0.6559 (5)	0.3380 (4)	0.6190 (4)	0.0368 (10)	
C4	0.8009 (5)	0.3151 (5)	0.5857 (4)	0.0488 (13)	
H4	0.8689	0.3117	0.6388	0.059*	
C5	0.8393 (6)	0.2978 (5)	0.4723 (5)	0.0556 (14)	
H5	0.9358	0.2835	0.4479	0.067*	
C6	0.7390 (6)	0.3008 (5)	0.3916 (5)	0.0586 (15)	
H6	0.7698	0.2883	0.3151	0.070*	
C7	0.5959 (6)	0.3221 (5)	0.4231 (4)	0.0498 (13)	
H7	0.5284	0.3240	0.3696	0.060*	
C8	0.5564 (5)	0.3404 (4)	0.5374 (4)	0.0366 (10)	
C17	0.3970 (5)	−0.0719 (5)	0.1933 (5)	0.0533 (14)	
H17A	0.4102	−0.0909	0.2726	0.080*	
H17B	0.4268	0.0067	0.1731	0.080*	
H17C	0.4533	−0.1281	0.1461	0.080*	
C18	0.2443 (5)	−0.0768 (4)	0.1750 (4)	0.0350 (10)	
C19	0.0187 (5)	−0.0157 (4)	0.1590 (3)	0.0312 (9)	
C20	−0.1133 (5)	0.0466 (4)	0.1514 (4)	0.0388 (11)	
H20	−0.1285	0.1256	0.1711	0.047*	
C21	−0.2197 (5)	−0.0141 (5)	0.1137 (4)	0.0475 (12)	
H21	−0.3098	0.0245	0.1087	0.057*	
C22	−0.1972 (5)	−0.1309 (5)	0.0826 (4)	0.0436 (12)	
H22	−0.2719	−0.1683	0.0558	0.052*	
C23	−0.0667 (5)	−0.1936 (4)	0.0904 (4)	0.0372 (10)	
H23	−0.0523	−0.2724	0.0698	0.045*	
C24	0.0422 (4)	−0.1341 (4)	0.1302 (3)	0.0290 (9)	
C16	0.1781 (6)	0.1346 (4)	0.2239 (4)	0.0428 (11)	
H16A	0.1005	0.1912	0.2050	0.051*	
H16B	0.2651	0.1594	0.1808	0.051*	
C14	0.1954 (5)	0.1390 (4)	0.3512 (4)	0.0336 (10)	
C15	0.2342 (5)	0.2456 (4)	0.3911 (4)	0.0350 (10)	
H15	0.2492	0.3096	0.3394	0.042*	
C10	0.2508 (5)	0.2582 (4)	0.5056 (4)	0.0364 (10)	
C11	0.2293 (6)	0.1621 (5)	0.5816 (4)	0.0541 (14)	
H11	0.2393	0.1692	0.6592	0.065*	
C12	0.1935 (7)	0.0564 (5)	0.5430 (4)	0.0638 (17)	
H12	0.1817	−0.0087	0.5941	0.077*	
C13	0.1748 (6)	0.0463 (5)	0.4277 (4)	0.0497 (13)	
H13	0.1478	−0.0251	0.4026	0.060*	
C9	0.2880 (5)	0.3757 (4)	0.5458 (4)	0.0412 (11)	
H9A	0.2934	0.4329	0.4812	0.049*	
H9B	0.2127	0.4048	0.6019	0.049*	

### Atomic displacement parameters (Å^2^)

**Table:**

	*U*^11^	*U*^22^	*U*^33^	*U*^12^	*U*^13^	*U*^23^
I1	0.0557 (2)	0.0445 (2)	0.0974 (3)	−0.01214 (17)	−0.0278 (2)	0.0134 (2)
I2	0.0586 (2)	0.0599 (2)	0.0386 (2)	−0.00834 (17)	0.00143 (15)	−0.00368 (16)
Cd1	0.03768 (19)	0.0418 (2)	0.03441 (19)	−0.00319 (15)	−0.00878 (14)	−0.00293 (15)
N1	0.031 (2)	0.043 (2)	0.0292 (19)	−0.0044 (16)	−0.0060 (15)	−0.0012 (16)
N2	0.037 (2)	0.038 (2)	0.034 (2)	−0.0047 (16)	−0.0103 (16)	−0.0060 (17)
N3	0.038 (2)	0.036 (2)	0.0288 (19)	−0.0082 (17)	−0.0047 (16)	−0.0063 (16)
N4	0.0314 (19)	0.0301 (19)	0.037 (2)	−0.0028 (15)	−0.0058 (15)	−0.0059 (16)
C1	0.037 (3)	0.047 (3)	0.045 (3)	−0.005 (2)	−0.005 (2)	−0.005 (2)
C2	0.035 (2)	0.029 (2)	0.031 (2)	−0.0043 (18)	−0.0064 (18)	−0.0024 (18)
C3	0.038 (3)	0.040 (3)	0.033 (2)	−0.004 (2)	−0.0036 (19)	−0.001 (2)
C4	0.038 (3)	0.063 (3)	0.046 (3)	0.001 (2)	−0.004 (2)	−0.001 (3)
C5	0.048 (3)	0.064 (4)	0.051 (3)	0.006 (3)	0.007 (3)	−0.002 (3)
C6	0.070 (4)	0.064 (4)	0.040 (3)	0.001 (3)	0.008 (3)	−0.013 (3)
C7	0.061 (3)	0.051 (3)	0.038 (3)	−0.003 (3)	−0.009 (2)	−0.007 (2)
C8	0.044 (3)	0.033 (2)	0.035 (3)	−0.007 (2)	−0.005 (2)	−0.004 (2)
C17	0.039 (3)	0.056 (3)	0.068 (4)	−0.011 (2)	−0.011 (3)	−0.006 (3)
C18	0.037 (2)	0.037 (2)	0.032 (2)	−0.009 (2)	−0.0052 (19)	−0.0003 (19)
C19	0.038 (2)	0.035 (2)	0.021 (2)	−0.0020 (19)	−0.0034 (17)	−0.0014 (18)
C20	0.047 (3)	0.035 (2)	0.032 (2)	0.001 (2)	0.001 (2)	−0.001 (2)
C21	0.031 (3)	0.059 (3)	0.050 (3)	0.000 (2)	−0.001 (2)	0.012 (3)
C22	0.030 (2)	0.058 (3)	0.044 (3)	−0.015 (2)	−0.007 (2)	0.009 (2)
C23	0.040 (3)	0.037 (3)	0.036 (3)	−0.011 (2)	−0.008 (2)	0.004 (2)
C24	0.027 (2)	0.033 (2)	0.027 (2)	−0.0050 (17)	−0.0050 (17)	0.0017 (18)
C16	0.060 (3)	0.036 (3)	0.035 (3)	−0.010 (2)	−0.010 (2)	−0.004 (2)
C14	0.034 (2)	0.034 (2)	0.034 (2)	−0.0060 (19)	−0.0029 (18)	−0.0029 (19)
C15	0.035 (2)	0.033 (2)	0.039 (3)	−0.0068 (19)	−0.0086 (19)	−0.001 (2)
C10	0.033 (2)	0.041 (3)	0.038 (3)	−0.006 (2)	−0.0105 (19)	−0.008 (2)
C11	0.078 (4)	0.059 (3)	0.029 (3)	−0.024 (3)	−0.009 (2)	−0.002 (2)
C12	0.102 (5)	0.057 (4)	0.037 (3)	−0.041 (3)	−0.005 (3)	0.005 (3)
C13	0.069 (4)	0.043 (3)	0.040 (3)	−0.026 (3)	−0.004 (2)	−0.004 (2)
C9	0.040 (3)	0.040 (3)	0.047 (3)	−0.001 (2)	−0.022 (2)	−0.008 (2)

### Geometric parameters (Å, °)

**Table:**

I1—Cd1	2.7121 (9)	C17—H17A	0.9600
I2—Cd1	2.7325 (10)	C17—H17B	0.9600
Cd1—N1^i^	2.275 (3)	C17—H17C	0.9600
Cd1—N4	2.294 (3)	C19—C24	1.392 (6)
N1—C2	1.314 (5)	C19—C20	1.391 (6)
N1—C3	1.389 (5)	C20—C21	1.368 (7)
N1—Cd1^i^	2.275 (3)	C20—H20	0.9300
N2—C2	1.367 (5)	C21—C22	1.382 (7)
N2—C8	1.385 (6)	C21—H21	0.9300
N2—C9	1.463 (5)	C22—C23	1.381 (6)
N3—C18	1.353 (6)	C22—H22	0.9300
N3—C19	1.377 (5)	C23—C24	1.389 (6)
N3—C16	1.457 (5)	C23—H23	0.9300
N4—C18	1.324 (5)	C16—C14	1.517 (6)
N4—C24	1.397 (5)	C16—H16A	0.9700
C1—C2	1.479 (6)	C16—H16B	0.9700
C1—H1A	0.9600	C14—C13	1.355 (7)
C1—H1B	0.9600	C14—C15	1.395 (6)
C1—H1C	0.9600	C15—C10	1.378 (6)
C3—C8	1.397 (6)	C15—H15	0.9300
C3—C4	1.391 (6)	C10—C11	1.382 (7)
C4—C5	1.362 (7)	C10—C9	1.503 (6)
C4—H4	0.9300	C11—C12	1.370 (7)
C5—C6	1.394 (8)	C11—H11	0.9300
C5—H5	0.9300	C12—C13	1.388 (7)
C6—C7	1.368 (7)	C12—H12	0.9300
C6—H6	0.9300	C13—H13	0.9300
C7—C8	1.377 (6)	C9—H9A	0.9700
C7—H7	0.9300	C9—H9B	0.9700
C17—C18	1.476 (6)		
			
N1^i^—Cd1—N4	95.46 (13)	N4—C18—N3	112.0 (4)
N1^i^—Cd1—I1	104.94 (9)	N4—C18—C17	125.0 (4)
N4—Cd1—I1	108.30 (9)	N3—C18—C17	122.9 (4)
N1^i^—Cd1—I2	113.68 (9)	C24—C19—N3	105.6 (4)
N4—Cd1—I2	99.05 (9)	C24—C19—C20	122.0 (4)
I1—Cd1—I2	129.68 (3)	N3—C19—C20	132.3 (4)
C2—N1—C3	106.6 (3)	C21—C20—C19	116.7 (4)
C2—N1—Cd1^i^	132.2 (3)	C21—C20—H20	121.6
C3—N1—Cd1^i^	121.1 (3)	C19—C20—H20	121.6
C2—N2—C8	107.5 (4)	C20—C21—C22	121.9 (4)
C2—N2—C9	128.1 (4)	C20—C21—H21	119.0
C8—N2—C9	124.4 (4)	C22—C21—H21	119.0
C18—N3—C19	107.8 (4)	C21—C22—C23	121.7 (4)
C18—N3—C16	126.0 (4)	C21—C22—H22	119.1
C19—N3—C16	126.2 (4)	C23—C22—H22	119.1
C18—N4—C24	105.5 (3)	C22—C23—C24	117.2 (4)
C18—N4—Cd1	126.5 (3)	C22—C23—H23	121.4
C24—N4—Cd1	128.0 (3)	C24—C23—H23	121.4
C2—C1—H1A	109.5	C19—C24—N4	109.1 (3)
C2—C1—H1B	109.5	C19—C24—C23	120.4 (4)
H1A—C1—H1B	109.5	N4—C24—C23	130.4 (4)
C2—C1—H1C	109.5	N3—C16—C14	114.1 (4)
H1A—C1—H1C	109.5	N3—C16—H16A	108.7
H1B—C1—H1C	109.5	C14—C16—H16A	108.7
N1—C2—N2	111.5 (4)	N3—C16—H16B	108.7
N1—C2—C1	125.4 (4)	C14—C16—H16B	108.7
N2—C2—C1	123.1 (4)	H16A—C16—H16B	107.6
C8—C3—C4	119.8 (4)	C13—C14—C15	118.5 (4)
C8—C3—N1	109.0 (4)	C13—C14—C16	124.1 (4)
C4—C3—N1	131.1 (4)	C15—C14—C16	117.5 (4)
C5—C4—C3	117.4 (5)	C10—C15—C14	121.5 (4)
C5—C4—H4	121.3	C10—C15—H15	119.2
C3—C4—H4	121.3	C14—C15—H15	119.2
C4—C5—C6	122.2 (5)	C15—C10—C11	118.7 (4)
C4—C5—H5	118.9	C15—C10—C9	119.9 (4)
C6—C5—H5	118.9	C11—C10—C9	121.4 (4)
C7—C6—C5	121.2 (5)	C12—C11—C10	120.3 (4)
C7—C6—H6	119.4	C12—C11—H11	119.9
C5—C6—H6	119.4	C10—C11—H11	119.9
C6—C7—C8	116.9 (5)	C11—C12—C13	120.1 (5)
C6—C7—H7	121.5	C11—C12—H12	120.0
C8—C7—H7	121.5	C13—C12—H12	120.0
C7—C8—N2	132.1 (4)	C14—C13—C12	120.9 (5)
C7—C8—C3	122.5 (4)	C14—C13—H13	119.5
N2—C8—C3	105.4 (4)	C12—C13—H13	119.5
C18—C17—H17A	109.5	N2—C9—C10	111.8 (4)
C18—C17—H17B	109.5	N2—C9—H9A	109.2
H17A—C17—H17B	109.5	C10—C9—H9A	109.2
C18—C17—H17C	109.5	N2—C9—H9B	109.2
H17A—C17—H17C	109.5	C10—C9—H9B	109.2
H17B—C17—H17C	109.5	H9A—C9—H9B	107.9
			
N1^i^—Cd1—N4—C18	43.2 (4)	C19—N3—C18—C17	177.2 (4)
I1—Cd1—N4—C18	151.0 (3)	C16—N3—C18—C17	−2.3 (7)
I2—Cd1—N4—C18	−71.8 (4)	C18—N3—C19—C24	0.5 (4)
N1^i^—Cd1—N4—C24	−135.6 (3)	C16—N3—C19—C24	180.0 (4)
I1—Cd1—N4—C24	−27.8 (3)	C18—N3—C19—C20	−176.6 (4)
I2—Cd1—N4—C24	109.4 (3)	C16—N3—C19—C20	2.9 (7)
C3—N1—C2—N2	0.4 (5)	C24—C19—C20—C21	−0.3 (6)
Cd1^i^—N1—C2—N2	−174.8 (3)	N3—C19—C20—C21	176.5 (4)
C3—N1—C2—C1	−178.8 (4)	C19—C20—C21—C22	−1.0 (7)
Cd1^i^—N1—C2—C1	5.9 (7)	C20—C21—C22—C23	1.4 (7)
C8—N2—C2—N1	−0.7 (5)	C21—C22—C23—C24	−0.4 (7)
C9—N2—C2—N1	178.9 (4)	N3—C19—C24—N4	0.2 (4)
C8—N2—C2—C1	178.6 (4)	C20—C19—C24—N4	177.7 (4)
C9—N2—C2—C1	−1.8 (7)	N3—C19—C24—C23	−176.3 (4)
C2—N1—C3—C8	0.0 (5)	C20—C19—C24—C23	1.2 (6)
Cd1^i^—N1—C3—C8	175.9 (3)	C18—N4—C24—C19	−0.8 (5)
C2—N1—C3—C4	178.9 (5)	Cd1—N4—C24—C19	178.2 (3)
Cd1^i^—N1—C3—C4	−5.2 (7)	C18—N4—C24—C23	175.2 (4)
C8—C3—C4—C5	1.1 (7)	Cd1—N4—C24—C23	−5.8 (6)
N1—C3—C4—C5	−177.7 (5)	C22—C23—C24—C19	−0.8 (6)
C3—C4—C5—C6	−0.9 (8)	C22—C23—C24—N4	−176.4 (4)
C4—C5—C6—C7	0.3 (9)	C18—N3—C16—C14	−74.6 (6)
C5—C6—C7—C8	0.2 (8)	C19—N3—C16—C14	106.1 (5)
C6—C7—C8—N2	178.4 (5)	N3—C16—C14—C13	−4.9 (7)
C6—C7—C8—C3	0.1 (7)	N3—C16—C14—C15	175.5 (4)
C2—N2—C8—C7	−178.0 (5)	C13—C14—C15—C10	−0.4 (7)
C9—N2—C8—C7	2.5 (8)	C16—C14—C15—C10	179.2 (4)
C2—N2—C8—C3	0.6 (5)	C14—C15—C10—C11	0.5 (7)
C9—N2—C8—C3	−179.0 (4)	C14—C15—C10—C9	−178.0 (4)
C4—C3—C8—C7	−0.7 (7)	C15—C10—C11—C12	0.5 (8)
N1—C3—C8—C7	178.4 (4)	C9—C10—C11—C12	179.1 (5)
C4—C3—C8—N2	−179.5 (4)	C10—C11—C12—C13	−1.7 (9)
N1—C3—C8—N2	−0.4 (5)	C15—C14—C13—C12	−0.8 (8)
C24—N4—C18—N3	1.2 (5)	C16—C14—C13—C12	179.6 (5)
Cd1—N4—C18—N3	−177.9 (3)	C11—C12—C13—C14	1.9 (9)
C24—N4—C18—C17	−177.1 (4)	C2—N2—C9—C10	−112.9 (5)
Cd1—N4—C18—C17	3.9 (6)	C8—N2—C9—C10	66.6 (6)
C19—N3—C18—N4	−1.1 (5)	C15—C10—C9—N2	−119.4 (5)
C16—N3—C18—N4	179.4 (4)	C11—C10—C9—N2	62.1 (6)

Symmetry codes: (i) −*x*+1, −*y*, −*z*+1.

## References

[bb1] Dobrzanska, L., Lioyd, G. O., Jacobs, T., Rootman, I., Oliver, C. L., Bredenkamp, M. W. & Barbour, L. J. (2006). *J. Mol. Struct.* **796**, 107–113.

[bb2] Li, J., Ji, C. C., Huang, L. F., Li, Y. Z. & Zheng, H. G. (2011). *Inorg. Chim. Acta*, **371**, 27–35.

[bb3] Raehm, L., Mimassi, L., Guyard-Duhayon, C., Amouri, H. & Rager, M. N. (2003). *Inorg. Chem.* **42**, 5654–5659. 10.1021/ic0340884 12950214

[bb4] Rigaku/MSC (2006). *CrystalClear* and *CrystalStructure* Rigaku/MSC, The Woodlands, Texas, USA.

[bb5] Sheldrick, G. M. (2008). *Acta Cryst.* A**64**, 112–122. 10.1107/S0108767307043930 18156677

[bb7] Wang, Y., Xu, H. B., Su, Z. M., Shao, K. Z., Zhao, Y. H., Cui, H. P., Lan, Y. Q. & Hao, X. R. (2006). *Inorg. Chem. Commun.* **9**, 1207–1211.

[bb8] Yu, X. Y., Zou, H. H., Wei, L. Q. & Zeng, M. H. (2010). *Inorg. Chem. Commun.* **13**, 1137–1139.

[bb9] Zhao, L.-Z., Li, P., Cao, B.-L. & Ng, S. W. (2009). *Acta Cryst.* E**65**, m613. 10.1107/S1600536809015943 PMC2969561 21582984

